# PDE4 as a target in preterm labour

**DOI:** 10.1186/1471-2393-7-S1-S12

**Published:** 2007-06-01

**Authors:** Céline Méhats, Thomas Schmitz, Stéphanie Oger, Roxane Hervé, Dominique Cabrol, Marie-Josèphe Leroy

**Affiliations:** 1 Institut National de la Santé et de la Recherche Médicale, INSERM U767, F-75270 Paris cedex 06, France; 2 Université Paris Descartes, F-75006 Paris, France; 3 AP-HP, Hôpital Cochin, Maternité Port-Royal, F-75014 Paris, France

## Abstract

Cyclic nucleotide phosphodiesterases (PDE) are the enzymes catalyzing the hydrolysis and inactivation of the second messengers, cAMP and cGMP. Eleven PDE families are described to date, and selective inhibitors of some PDEs families are currently used in clinic for treating cardiovascular disorders, erectile dysfunction, and pulmonary hypertension. Isoforms of the PDE4 family are involved in smooth muscle contraction and inflammation. PDE4 selective inhibitors are currently in clinical trials for the treatment of diseases related to inflammatory disorders. Because of their myorelaxant properties, we first examined their expression in human myometrium and uncover an increased expression of one specific isoform, PDE4B2, in the near-term myometrium as compared to myometrium in the nonpregnant state. Using human myometrial cells in culture, we demonstrated that PDE4B2 can be induced by its own substrate, under the control of one of the major utero-contractile agonists, PGE_2_, itself upregulated by the proinflammatory cytokine IL-1β. Functionally, augmentation of global PDE4 activity decreases the ability of β-adrenergic agonists (the most commonly used tocolytic drugs) to inhibit myometrial contraction at the end of pregnancy and during pathophysiological situations, such as persistent intrauterine inflammation which is a major cause of very preterm delivery. Currently exploring the anti-inflammatory properties of PDE4 inhibitors in gestational tissues, we recently demonstrated the ability of these drugs to block a persistent inflammatory response of the foetal membranes in Humans and to prevent inflammation-driven preterm delivery and foetal demise in mice. These data open up a new therapeutical strategy to prevent inflammation-induced preterm delivery and its sequelae in very preterm infants.

## Background

Tea leaf has been used for 2,000 years in traditional Chinese medicine for its therapeutical virtues before becoming a traditional drink. One of its main active ingredients, theophylline, is a selective inhibitor of cyclic nucleotide phosphodiesterases (PDE), which hydrolyses the second messengers, cAMP and cGMP. Eleven families of PDE are described to date with different substrate preference, kinetics and modulators [1]. Selective PDE inhibitors of the type 3 and type 5 families are currently used in clinic, respectively for cardiac disease, erectile dysfunction, and pulmonary hypertension. PDE4 specifically hydrolyses cAMP with high affinity and PDE4 inhibitors are highly scrutinized because of their myorelaxant and anti-inflammatory properties.

**Figure 1:**
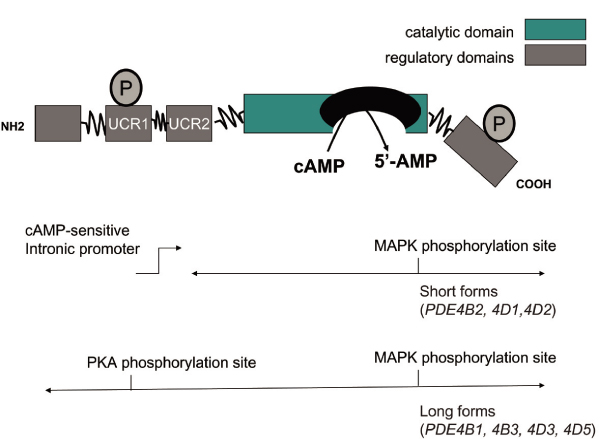
**Schematic organization of the PDE4 family**. PDE4s have a highly conserved globular catalytic domain, flanked by regulatory domains, such as the PDE4 signature domains UCR1 and UCR2 (for Upstream Conserved Regulatory domains), containing, for example, a PKA phosphorylation site. PDE4s are divided in short or long forms, defined by the absence or the presence of UCR1. Some examples of short and long forms, expressed or regulated in human myometrium are quoted. Short form products of PDE4B and PDE4D gene derived from a cAMP-sensitive intronic promoter. Long forms are usually constitutive.

PDE4 is the largest PDE family known to date, encoded by four distinct genes, PDE4A-D, and generating more than 20 different isoforms in mammals by means of differential promoters and splicing. The PDE4s are further divided in two main classes, the long and short forms that differ with the presence or not of regulatory domains at their amino-terminus [2,3] (Figure 1). In particular, the presence of a PKA-phosphorylation site well-conserved over the PDE4 long forms activates these isoforms in a short-term feedback loop. Conversely, cAMP- and hormonal-sensitive promoters control the transcription of some of the PDE4 short-forms, leading to a decrease in cAMP levels after a sustained elevation of the cyclic nucleotide content. These regulations, as we describe below, are critical to lessen the cAMP signalling in the utero-placental interface at the end of pregnancy.

## Results

**Figure 2:**
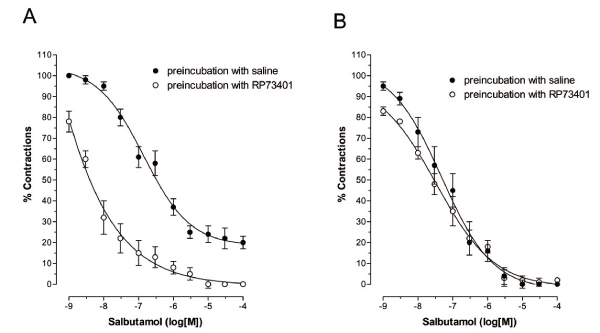
**Potentiation of the relaxant effect of β-adrenergic agonist in the presence of a PDE4 selective inhibitor in pregnant myometrium**. Strips were mounted for isometric recording under 900 mg tension and after 2 h equilibration, contractile activity was assessed. Myometrial strip of either pregnant **(A) **or nonpregnant women **(B)** were preincubated with 3 × 10^-9^M RP73401, a competitive selective PDE4 inhibitor (open circles) or saline (closed circles), 30 min prior the construction of the concentration-response to salbutamol. Results are expressed in percentage of maximal contraction at the end of the equilibration period. Strips from the same patients were subjected in parallel to treatments. Data are expressed as the mean ± SEM for strips isolated from myometrium of five different pregnant women and three different nonpregnant women. (Adapted from [8]).

We examined in human myometrium the expression of the four genes of PDE4 in the nonpregnant state and near term. A concurrent expression of the mRNAs of the four genes with a predominance of the PDE4D and PDE4B mRNAs and a weaker expression of PDE4A and PDE4C genes was observed. Using semi-quantitative PCR and Western blot, we observed, in near term myometrium, an increase of the steady-state levels of PDE4B2 mRNA [4] and protein [5], a short form product of the PDE4B gene. We also explored functionally whether PDE4s are involved in the control of the myometrial contractility. Selective PDE4 inhibitors of the first (rolipram) and of the most recent developed generation achieve a complete relaxation of the myometrial strips, either in nonpregnant or pregnant state [4-6]. Although the β-adrenergic agonists, the most commonly used tocolytic agents, are able to completely relax the nonpregnant myometrium, they are unable to fully inhibit the pregnant uterine contractions. This phenomenon reflects the desensitization of the β-adrenergic system at the end of the pregnancy [7]. Application of a PDE4 inhibitor potentiates the effect of β-mimetics, which then achieves a complete relaxation of the pregnant strip [8] (Figure 2). It is worth noting that PDE4 inhibitors do not potentiate the effect of or act in synergy with β-adrenergic agonists on nonpregnant strips, indicating an increased role of the PDE4s in the contractility of the pregnant state.

We explored in parallel the molecular mechanism of the hormonal regulation of the PDE4 expression in primary culture of myometrial cells. Like in the tissue of origin, PDE4 is the major cAMP-hydrolyzing activity in these cells and the four PDE4 genes are expressed. An increase in intracellular cAMP concentration led to a significant increase in cAMP PDE activity through the selective induction of the short forms of the PDE4B and PDE4D genes, namely PDE4B2, PDE4D1 and PDE4D2 [9]. Hence, PDE4 upregulation provides a feedback mechanism by which cells might adapt from sustained stimulation of the cAMP pathway. We then shown that prostaglandin E_2_, one of the major effectors of labour, can induce PDE4B2 and PDE4D short forms, through a cAMP-dependent pathway, in the myometrial smooth muscle cells [8]. This regulation may occur during labour, when PGE_2 _concentration rises dramatically, but also in certain pathophysiological situations, with intrauterine inflammation, because we observed that the proinflammatory cytokine IL1β, by increasing PGE_2_concentration, is also able to induce PDE4B2 [10]. Furthermore, impregnation of myometrial strips with PGE_2 _leads to a heterologous desensitization of the β-adrenergic system. This is translated by a decrease of the relaxant strength of β-adrenergic agonists and is reversed in the presence of PDE4 inhibitors [8]. Therefore, a co-application of β-mimetics and PDE4 inhibitors may greatly improve the efficiency of the tocolysis in case of preterm labour.

Besides, because PDE4 activity is high in the amnion and the chorion, we established that not only the myometrium is a target for PDE4 inhibition but also the fetal membranes. These data are of importance because amniochorionic membranes are major sources of pro-inflammatory mediators and uterotonic agents. Indeed, treatment of amniochorionic explants with the membrane product of *E. coli*, the lipopolysaccharide (LPS), induced synthesis of a pro-and anti-inflammatory cytokine, TNFα and IL-10 respectively but also induced synthesis of prostaglandins (PGs) and metalloproteases (MMPs) [11]. In addition, PDE4 activity was independently increased by LPS treatment. On this model of explants, we demonstrated that PDE4 selective inhibitors diminished greatly the production of TNFα whereas they enhanced the release of IL-10. They then decreased the production of PGE_2 _and MMP9. Recently, we derived primary cells from the different cell types present in amniochorionic membranes: amniotic epithelial cells, fibroblasts and chorionic epithelial cells, the choriotrophoblasts, which serve as models to identify signaling pathways involved in a local inflammation.

**Figure 3:**
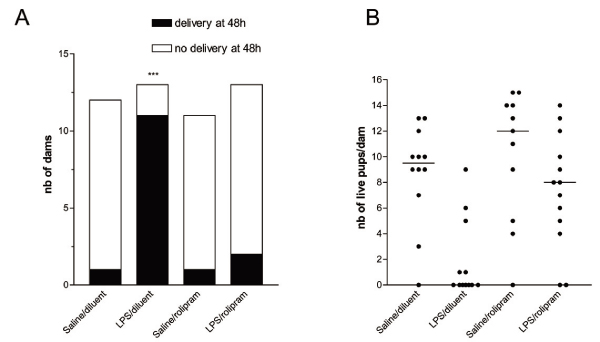
**Inflammation-induced preterm delivery is prevented by rolipram**. Delivered and undelivered dams were numbered 48 h after intrauterine injection of LPS or saline **(A) **and number of live pups per dams were recorded **(B)**. The Fisher exact test was applied for comparison of preterm delivery rate in each group. ***p < 0.001 versus saline-vehicle group. For comparison of the number of live per dam, statistical difference between groups was assessed by two-way ANOVA, followed by Student's *t *test, two tailed for unpaired samples.^†††^p < 0.001, versus the saline-vehicle group. (Adapted from [12]).

Very recently, we demonstrated that PDE4 inhibition prevents *in vivo *preterm delivery and fetal demise in mice [12]. We adapted the inflammation-induced preterm delivery model in mice established by Elovitz *et al. *[13]. On day 15 of gestation (term 20 days), LPS or saline was injected in the uterus. Two hours after surgery, vehicle or the PDE4 selective inhibitor, rolipram, was injected in the peritoneal cavity and the frequency of preterm delivery and the fetal demises were recorded 48 h post-surgery. In the presence of rolipram, we observed that LPS-induced preterm delivery is prevented as well as fetal demise, the number of live pups in rolipram-LPS injected mice being not statistically different from the control group (Figure 3). Moreover, we showed that the increased cytokine concentrations in the amniotic fluid of LPS-treated dams were significantly reduced by the treatment with rolipram. We evaluated by immunohistochemistry whether the transcription factor of inflammation, NFκB, was translocated in the nucleus in gestational tissues as an index of activation. Interestingly we found that, six hours after LPS injection, NFκB was located in the nucleus of a specific placental cell, at the feto-maternal interface, the glycogen trophoblast whereas this translocation was absent in trophoblasts of dams treated with rolipram. No nuclear translocation of NFκB was observed in myometrial cells.

## Conclusion

**Figure 4:**
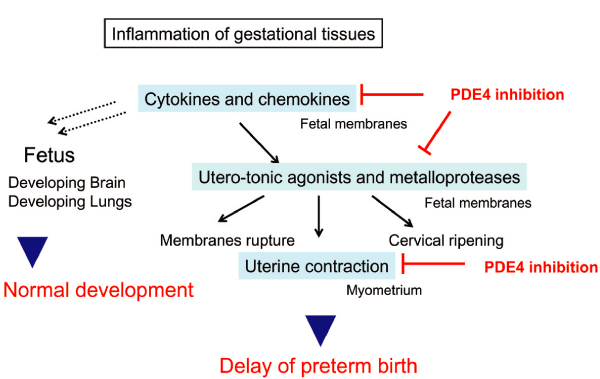
**Summary of the effects of the PDE4 inhibition in gestational tissues in the case of intra-uterine inflammation**. Intrauterine inflammation leads to massive production of cytokines and chemokines, mainly by the fetal membranes, the chorio-decidua. Cytokines and chemokines start a feedforward mechanism and the production of uterotonic agonists and metalloproteases in the chorio-decidua. These agents further digest the fetal membranes, contract the myometrium, and soften the cervix, causing preterm labor. Moreover, inflammation of the amniotic cavity may target the developing brain and lungs of the fetus. We showed that PDE4 inhibition may directly in the human myometrium and indirectly by decreasing production of cytokines in the fetal membranes diminish uterine contraction and delay preterm delivery. Furthermore, by decreasing intrauterine inflammation, PDE4 inhibitors may favor a normal development of the fetal brain and lungs.

These original studies uncover a new potential target, the myometrial PDE4s, for tocolysis. In addition, PDE4 inhibitors are able to block the production of inflammatory cytokines produced by myometrial and fetal explants that may induce preterm delivery (Figure 4). Moreover the *in utero *exposure to inflammation is a high risk factor of neurological and respiratory disorders of the preterm infant. Therefore, the combination of the myorelaxant and anti-inflammatory properties of the PDE4 inhibitors make these molecules particularly attractive for the prevention of inflammation-induced preterm delivery and its consequences on the premature infant.

## Competing interests

The authors declare that they have no competing interests.
